# Clinical Evaluation of Li Brush Endometrial Samplers for Diagnosing Endometrial Lesions in Women With Intrauterine Devices

**DOI:** 10.3389/fmed.2020.598689

**Published:** 2020-11-30

**Authors:** Lu Han, Sijia Ma, Lanbo Zhao, Yu Liu, Yiran Wang, Xue Feng, Kailu Zhang, Lei Wang, Li Wang, Panyue Yin, Dongxin Liang, Huilian Hou, Guizhi Shi, Qiling Li

**Affiliations:** ^1^Department of Obstetrics and Gynecology, The First Affiliated Hospital of Xi'an Jiaotong University, Xi'an, China; ^2^Health Science Center, Xi'an Jiaotong University, Xi'an, China; ^3^Department of Pathology, The First Affiliated Hospital of Xi'an Jiaotong University, Xi'an, China; ^4^Aviation General Hospital of Beijing, Medical University & Beijing Institute of Translational Medicine, University of Chinese Academy of Sciences, Beijing, China

**Keywords:** endometrial lesions, cytology, intrauterine devices, li brush, cancer screening

## Abstract

**Background:** For women with intrauterine devices (IUDs), it is difficult to sample the endometrium when abnormal uterine bleeding occurs or when regular screening of endometrial cancer is proposed. The purpose of this study is to evaluate the validity of endometrial sampling using Li Brush in IUD users.

**Methods:** This study was a prospective cohort study and conducted in two parts. Part I was to assess the impact of Li Brush on the position of IUDs. Transvaginal ultrasound was used to locate IUDs before and after sampling. Part II was to explore the diagnostic accuracy of Li Brush in detecting endometrial lesions. IUD users with irregular uterine bleeding were recruited in the IUD group and IUD non-users who arranged for dilatation and curettage (D&C) were recruited in the control group. The endometrium was sampled by Li Brush for cells and by D&C for tissues in both groups. The satisfactoriness of sampling and validity of Li Brush were evaluated.

**Results:** Seventeen cases in part I confirmed no significant difference in the position of IUDs before and after sampling (*p* = 0.20). 112 IUD users and 139 IUD non-users were recruited in part II. Li Brush achieved 94.64 and 92.09% satisfactory sampling rates in the IUD group and control group, respectively, without statistically significant difference between the two groups (*p* = 0.42). The Sensitivity and specificity of Li Brush for detection of endometrial lesions in IUD group were 95.35 and 87.76% respectively.

**Conclusions:** Li Brush used for endometrial biopsy did not affect the position of IUDs and had high yield of satisfactory samples and good validity for endometrial diagnoses. It was feasible to screen endometrial lesions by Li Brush for women with IUDs.

## Background

Intrauterine devices (IUDs) are widely used and effective long-acting reversible contraception. They effectively reduce the incidence of unintended pregnancies (1), and have been applied increasingly during the past decade, from 5.8% in 2008 to 11.8% in 2014 in the US (2), and 52.3% in 2007 in China (3). IUDs are of two types: copper intrauterine devices (Cu-IUDs) and levonorgestrel intrauterine systems (LNG-IUSs), with 0.8% and 0.2% failure rates, respectively (4). Cu-IUDs are recommended as the preferred long-term contraceptive method for postpartum (5), breastfeeding (6), and perimenopausal women, and even for immediate placement after a surgical abortion (4). LNG-IUSs, in addition to the aforementioned uses, are also recommended for conservative treatment of endometrial hyperplasia (7) (including atypical hyperplasia) and early endometrial cancer (8). In China, there are currently eight types of IUDs employed clinically, including one LNG-IUS named Mirena and seven copper intrauterine devices (OCu200, HCu280, Cu365, MLCu375, MYCu, GyneFix IN, and TCu220c) (9).

Endometrial lesions are commonly encountered in a gynecological clinic, and irregular vaginal bleeding often presents as the first symptom of women with endometrial lesions (10). When endometrial lesions occur, the pathological features play a key guiding role in clinical treatment. Consequently, endometrial biopsy by dilatation and curettage (D&C) is required in these patients for pathological diagnosis before treatments (11). The difficulties and obstacles arise when an endometrial biopsy is required in an IUD user who has irregular vaginal bleeding, especially under the screening circumstance of endometrial cancer, because the IUD needs to be removed before the D&C. This procedure may result in mental stress and economic loss for most of the women. Those with benign endometrial lesions must have the IUD re-inserted for long-term contraception. Moreover, regular supervision of the endometrium is also impossible for women conservatively treated by LNG-IUSs, or young breast cancer patients requiring contraception and tamoxifen therapy (12).

The disposable endometrial sampler, Li Brush, is highly effective in the screening of endometrial lesions. The sensitivity and specificity for screening endometrial cancers and precancerous lesions were 92.73 and 98.15%, respectively (13–15). The high accuracy of Li Brush also has been confirmed for the diagnosis of different endometrial conditions in previous studies (14).

The purpose of this study is to investigate the validity of endometrial sampling using Li Brush in women with IUDs. This study was conducted in two parts. Part I was to assess the impact of endometrial cytology using Li Brush on the position of IUDs, and part II was to explore the diagnostic accuracy of Li Brush in detecting endometrial lesions.

## Materials and Methods

### Patients

We recruited women with IUDs in the Outpatient Department of Gynecology. The premenopausal IUD users with prolonged menstrual period, irregular vaginal bleeding, or spotting were enrolled in part I from December 2018 to March 2019. These were women who refused to undergo curettage and initially required medical treatments. For part II, IUD users and IUD non-users who were scheduled for D&C due to abnormal uterine bleeding or postmenopausal bleeding were selected from April 2019 to January 2020. Women with IUD served as the experimental cohort, and women without IUD served as the control group. The sample size was calculated using PASS 11.0.4. Part I enrolled at least 10 women (α = 0.05, 1-β = 0.80, δ = 0.10, Mean of Paired Differences = 0.1, *S* = 0.12), and each group in part II included at least 78 women (α = 0.025, 1-β = 0.80, δ = −0.10). Moreover, regardless of part I or part II, women were excluded if they had acute inflammation of the reproductive system, cervical cancer, suspected pregnancy, or other surgical contraindications. All IUD users participating in this study required the normal positions of IUDs.

For all participants, the following information was collected: age, last menstrual period or menopausal age, fertility history, endometrial thickness, type of IUD, and time since IUD insertion. In addition, the positions of IUDs were required for part I, and the endometrial diagnoses were required for part II.

Ethical approval for this clinical study was obtained by the Ethics Committee of the First Affiliated Hospital of Xi'an Jiaotong University (XJTU1AF2018LSK-042), and the clinical trial was registered (ChiCTR1800020123). All patients involved in this project sufficiently comprehended the research and provided informed consent.

### Study Procedures

For part I, endometrial biopsies were performed by Li Brush (Xi'an Meijiajia Bio-Technologies Co. Ltd., China, 20152660054) after the normal position of IUDs were confirmed by transvaginal ultrasonography which has been found to be effective in locating IUDs (16, 17). The sampling procedure of Li Brush was performed according to the protocol in the previous article (14). The distance from the upper edge of the IUD to the uterine fundus was measured before and after sampling. The validity of endometrial biopsy by Li Brush in IUD users was evaluated by comparing the two positions of IUDs before and after sampling.

For part II, an endometrial sampling of each participant was performed by Li Brush before D&C or removing the IUD. Specific solutions were used to preserve specimens on the bristles of Li Brush (18). The specimens were centrifuged at 3,000 rpm for 3 min. The cellular components at the bottom were used to make smears and stained with hematoxylin-eosin (19). Subsequently, the endometrial biopsies were performed by D&C in both IUD users (after removing IUDs) and IUD non-users. Li Brush sampling was performed by a single operator. Although D&C was performed by different operators, all operators received training from the same medical center. The tissues were embedded in paraffin and stained with hematoxylin-eosin. The paraffin section and cytological smear of each participant were diagnosed independently by two pathologists. The validity of Li Brush for diagnosing different endometrial conditions was explored by comparing histopathological and cytopathological diagnoses of individuals in the two groups. The flow chart for the whole study is in Supplementary Figure 1.

### Sonographic and Pathologic Criteria

The evaluation criteria of ultrasound were as follows: (1) the distance from the upper edge of the IUD to the serosa layer of the uterine fundus was no more than 2 cm (Supplementary Figures 2A,C); (2) The upper edge of the IUD fell on the center line in the longitudinal section of the uterus (Supplementary Figure 2B); (3) for Mirena IUDs, all three features were clearly visible in the ultrasound, including two echogenic dots corresponding to the two extremities and a shadow of the vertical shaft, but the best appropriate distance from its upper extremity to the uterine fundus was half of the anteroposterior diameter of the uterus; and (4) for a larger uterine size, the normal positions were evaluated by locating both upper and lower edges of the IUD (20, 21).

Satisfactory cytological specimens conformed to the following standards: (1) completed clinical information; (2) sufficient and well-preserved endometrial glandular epithelial cells, which required at least 10 piles and 5 piles of endometrial glandular epithelial cells in reproductive-aged women and postmenopausal women with atrophic endometrium, respectively; (3) The specimens with abnormal cells (atypical hyperplastic cells and malignant cells) were identified as satisfactory samples (22). Satisfactory histological specimens required effective fixation, no contamination by blood or impurities, sufficient endometrial glands, and epithelium for pathological diagnoses (23).

According to the new Bethesda-style classification, the cytopathological diagnoses of specimens obtained by Li Brush in this study were divided into the following categories: (1) unsatisfactory; (2) negative endometrial cells characterized by proliferative, secretory, atrophic, or mixed endometrial cells; (3) endometrial hyperplasia without atypia; (4) atypical endometrial cells with undetermined significance; (5) atypical endometrial cells; and (6) malignant cells (24). To correspond with the cytological system, the histopathological diagnoses of endometrial tissue obtained by D&C were classified into the following categories: (1) benign endometrium, which was further identified as proliferative, secretory, atrophic, mixed endometrium, or endometritis; (2) non-atypical endometrial hyperplasia; and (3) atypical hyperplasia and carcinoma (25). Positive results were defined as all lesions requiring further treatment, such as endometrial carcinoma, endometrial atypical hyperplasia, and endometrial hyperplasia without atypia. Other categories were defined as negative results. For diagnostic accuracy, an accurate case was defined as when the category of the cytological diagnosis was exactly same as the histological examination; otherwise, it was defined as an inaccurate case.

### Statistics

In this research, the statistical analysis was conducted using SPSS 23.0. The age of the participants was described by arithmetic mean and standard deviation. The median was used to describe the time since IUD insertion. For part I, the normally distributed data of IUD positions were described by arithmetic mean and standard deviation. The difference between two sets before and after sampling was measured using two related-sample Wilcoxon rank-sum tests. For part II, the age of participants in the two groups was compared by a Student *t* test. The menopausal status, time since IUD insertion, and endometrial thickness between the two groups were counted using the chi-square test and rank-sum test. The validity was evaluated by the following indicators: sensitivity (Se), specificity (Sp), false-negative rate (FNR), false-positive rate (FPR), positive predictive value (PV+), and negative predictive value (PV-). Satisfactory sampling rate of Li brush or D&C was defined as the proportion of satisfactory specimens. The validity of Li Brush was evaluated by McNemar chi-square or chi-square test and by Fisher exact test for the subtypes of IUDs. The distinctions of satisfactory sampling between Li Brush and D&C, or the IUD group and the control group, were assessed using the McNemar chi-square test. All calculated *p*-values were two-sided. The statistical significance level was set at *p* < 0.05.

## Results

**Figure 1:**
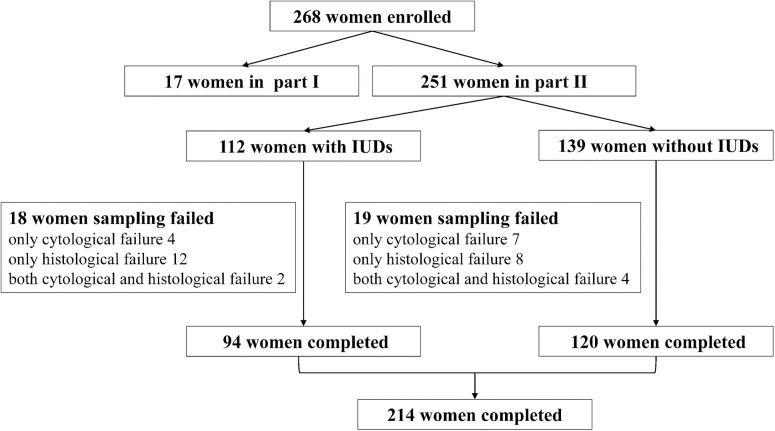
Diagram of study participants.

**Figure 2:**
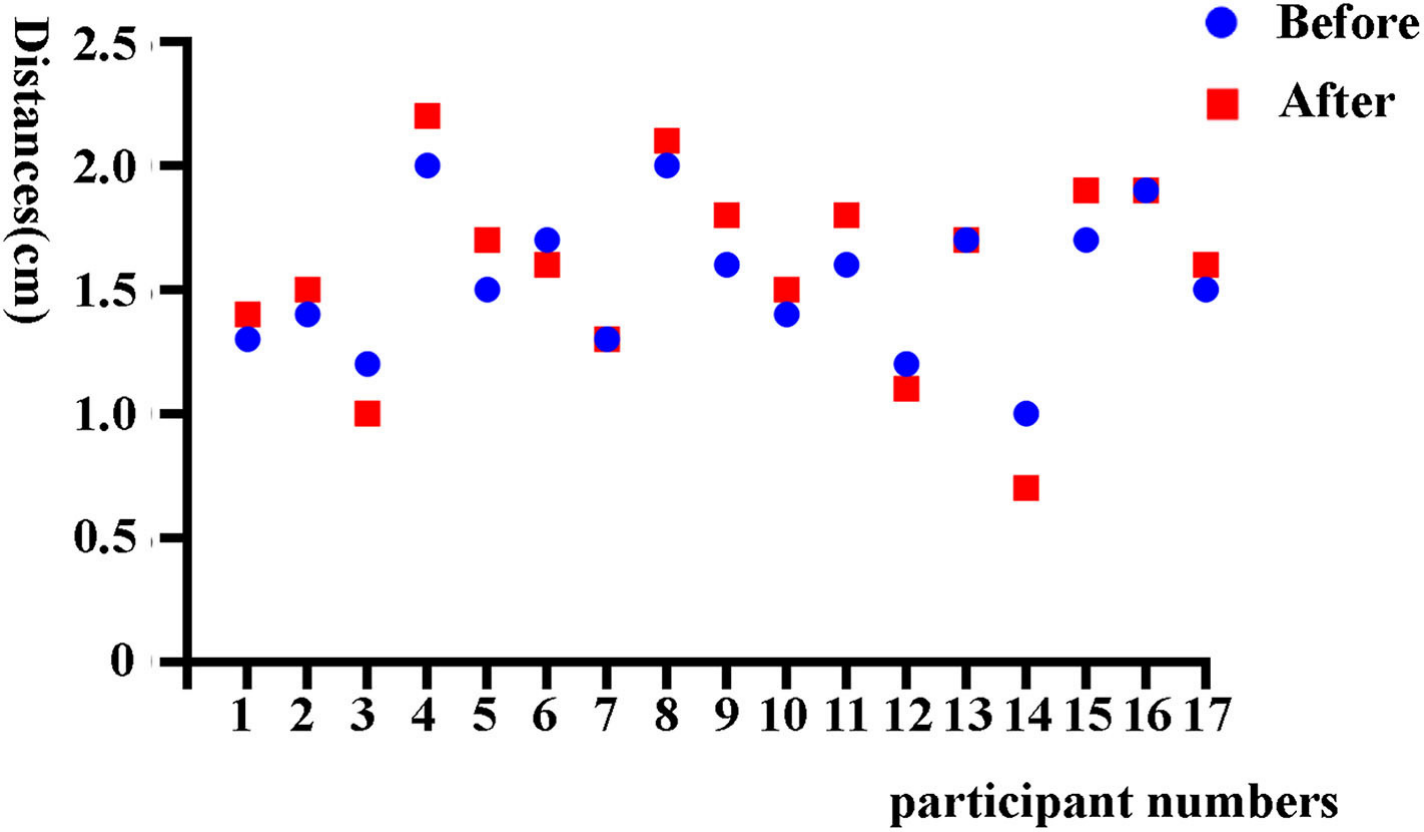
The positions of IUDs before and after sampling in part I.

A total of 268 women were recruited in this study, including 17 women in part I and 251 women in part II (Figure 1). In part I, the mean age of 17 participants was 43.53 ± 8.21 years, and the median time since IUD insertion was 9 years. The group had primarily six types of IUDs, including four OCu200, one MLCu375, one MYCu, two GyneFix IN, one TCu220c, four LNG-IUS, and four others. The positions of IUDs before and after sampling were 1.53 ± 0.29 cm and 1.58 ± 0.39 cm, respectively (Figure 2), which indicated no statically significant difference (*p* = 0.20). Consequently, Li Brush sampler for endometrial biopsy had no effect on the position of IUDs.

In part II, the entire cohort was comprised of 251 women aged 25 to 65 years, including 112 IUD users and 139 IUD non-users. The average age of women in this cohort was 45.13 ± 6.52 years in the IUD group and 45.80 ± 6.31 years in the control group. No significant difference was found in the age (*p* = 0.41) and menopausal status (*p* = 0.58) between the two groups (Table 1). Two hundred fourteen of 251 (85.26%) cases were diagnosed by both histological and cytological examinations. The diagnoses of 4.38% cases were based on histological sections alone. The diagnoses of 7.97% cases were made by cytological smears only. In addition, there were six cases that failed in both cytological and histological examinations. Ultimately, Li Brush for endometrial sampling achieved an overall satisfactory sampling rate of 93.23%, of which the IUD group was 94.64% and the control group was 92.09%. The overall satisfactory sampling rate of D&C was 89.64%, which was 87.50 and 91.37% in the two groups, respectively (Table 2). There was no significant difference in satisfactory sampling rates of Li Brush between the two groups (*p* = 0.42).

**Table 1:** Characteristics of participants in part II.

	**IUD group (*N* = 112)**	**Control group (*N* = 139)**	***P*-value**
Age (y)	45.13 ± 6.52	45.80 ± 6.31	0.41
Menstruation (*n*)			0.58
Menopause	19	20	
Premenopausal	93	119	
Endometrial thickness (*n*)			<0.01
<5mm	16	9	
≥5mm	85	130	
Abnormal echo	1	0	
Unclear display	1	0	
Unmeasured	9	0	
Time since IUD insertion (y)	10	-	
Type of IUD (*n*)			
OCu200	26	-	
MYCu	35	-	
GyneFix IN	3	-	
Cu365	21	-	
HCu280	5	-	
MLCu375	7	-	
TCu220c	8	-	
LNG-IUS	1	-	
others	6	-	

**Table 2:** Sampling Quality of Li Brush sampler and curettage.

**Li Brush**	**D&C**		**Total**
	**Satisfactory**	**Unsatisfactory**	
Satisfactory	214(94:120) ^*^	20(12:8)	234(106:128)
Unsatisfactory	11(4:7)	6(2:4)	17(6:11)
Total	225(98:127)	26(14:12)	251(112:139)

* *(IUD group: control group)*.

In part II, there were 93 true-positive, 105 true-negative, 10 false-positive, and 4 false-negative cases (Table 3). In IUD group, the following indices were obtained: Se 95.35%, Sp 87.76%, FNR 4.65%, FPR 12.24%, PV+87.23%, and PV −95.56%. In control group, the outcomes were Se 96.30%, Sp 93.94%, FNR 3.57%, FPR 6.06%, PV+92.86%, and PV −96.88%.

**Table 3:** The validity of Li Brush in detecting endometrial lesions.

**Li Brush**	**D&C**		**Total**
	**Positive**	**Negative**	
Positive	93(41:52)^a^	10(6:4)	103(47:56)
Negative	4(2:2)	105(43:62)	109(45:64)
Total	97(43:54)	115(49:66)	212^b^

a *(IUD group: control group)*;

b *The histological outcomes of two patients reported only polyps but had no specific features of the endometrium for the comparison in the IUD group*.

**Figure 3:**
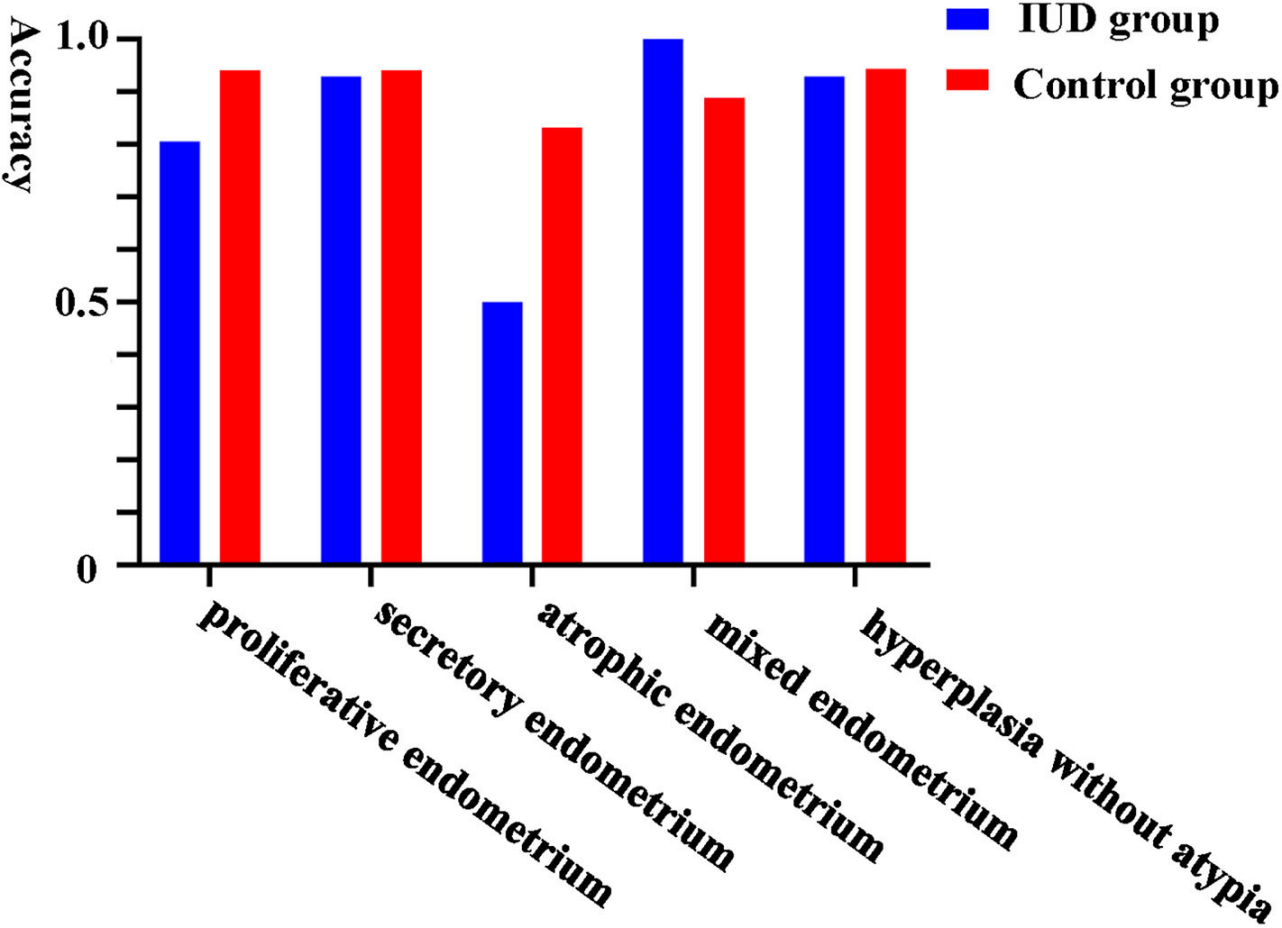
Comparison of diagnostic accuracy of Li Brush between the two groups in part II.

Furthermore, we evaluated the validity of using Li Brush to diagnose the different endometrial subtypes in two groups. There were 193 cases concordant in histopathological and cytopathological diagnoses, including 81 cases in the IUD group and 112 cases in the control group. The diagnostic accuracy of Li Brush was 88.04% in the IUD group and 93.33% in the control group, which demonstrated no distinction (*p* = 0.18) (Supplementary Table 1). The accuracies in different endometrial conditions of the two groups were 80.65 and 94.12% for proliferative, 92.86 and 94.12% for secretory, 50.00 and 83.33% for atrophic, 100.00 and 88.89% for mixed endometrium, and 92.86 and 94.44% for hyperplasia without atypia, respectively (Figure 3). For the subtypes of IUDs, no statistical significance was evident in the diagnostic accuracy of Li Brush (*p* = 0.81) (Supplementary Table 2). Taken together, the existence of IUDs did not affect the validity of Li Brush sampler in the endometrial cytology. However, high accuracies were obtained in all except for atrophic endometrium, because only two cases with atrophic endometrium were recruited in the IUD group. The effectiveness of Li Brush in atrophic endometrium needs to be verified by more cases.

## Discussion

Screening endometrial lesions has been a research hotspot, especially for women with risk factors for endometrial cancer (26). Recently, research studies have focused on more minimally invasive screening devices and detecting methods (13, 27). Nevertheless, no satisfactory screening method existed for women with IUDs. In this study, we first proposed Li Brush sampler for endometrial sampling in women with IUDs. Part I of the study involved six types of IUDs and part II involved eight types, all of which were commonly used for long-acting contraception. The positions of IUDs were not shifted by Li Brush, and the pathological diagnoses of endometrial lesions were also quite accurate in women with different types of IUDs.

Due to the specific structure of Li Brush, the T-shaped head without ring and hard-barbed structures, the position of an IUD is not shifted during sampling. The 3-mm-diameter elastic drive-pipe easily accesses the uterine cavity. And, owing to the flexible, soft bristles of Li Brush, IUDs are not deformed. Further, the fusiform brush and dense bristles guarantee adequate sampling of the uterine cavity (14).

There were 11 cases diagnosed inaccurately by Li Brush in the experimental group in part II. The endometrial lesions of 72.73% (8/11) cases were overly diagnosed by cytopathological examinations. Among them, two negative cases were misdiagnosed as atypical hyperplasia and four negative cases were misdiagnosed as benign hyperplasia by Li Brush. In addition, two positive women with endometrial simple hyperplasia were inaccurately diagnosed as complex endometrial hyperplasia and atypical hyperplasia. One possible reason was the incomplete sampling of local intrauterine lesions in D&C (28), and the other was the effect of IUDs on the endometrial diagnosis.

IUDs are known to produce a sterile foreign-body inflammatory reaction within the uterine cavity, especially the tissues around the IUDs, that enhances the contraceptive effect (29). The characteristics of foreign-body reaction are a significant increase in the number of neutrophils, monocytes, and plasma cells (30). For Cu-IUDs, the copper released from the IUD is oxidized by enzymes and then dissolved by the amino acids in the endometrial fluid, which alters the endometrial receptivity and immune response (31) and influences the metabolism of endometrial cells (30). In addition, Cu-IUDs induce cytokine production to aggregate immune cells in the endometrium, strengthening the inflammatory response (32, 33). For LNG-IUS, the high concentration of levonorgestrel causes the morphological change of endometrium with massive stromal decidualization and glandular atrophy (34). These biomedical mechanisms produce both histological and cytological changes in the endometrium, which should be considered during the endometrial diagnosis.

Regarding the histopathological changes, the surface endometrial tissues around IUDs showed necrosis with extensive leukocyte infiltration including neutrophils and plasma cells (35). Besides, the stimulation of an IUD leads to local proliferative changes, endometrial metaplasia, and subepithelial lymphocytic infiltration (33, 36). For cytological diagnoses, the normal endometrium of women with IUDs needs to be distinguished from the endometritis and benign hyperplasia. The cytological diagnoses in this research were based on the reporting format proposed by Yanoh K et al. and the Yokohama system (24, 37). However, neither diagnostic criteria specifically display the identifications for the cytological diagnosis of endometrium with IUDs. Based on the histochemical features of endometrium with IUDs, we summarized the following experiences for cytopathological diagnoses in this study: (1) the background in the cytological smears of normal endometrium with IUDs was clean and pure with the lymphocytic infiltration and a small number of neutrophils; (2) the background in the case of endometritis was dirty and complicated with lymphocytic infiltration, numerous inflammatory cells, histiocytes, and plasma cells; (3) local endometrial cells around the IUD sometimes appeared as simple hyperplasia or even complex hyperplasia, which lead to misdiagnosis of cytology; this misdiagnosis can be decreased by combining medical history, using cell block technique and, if necessary, by further histological test; (4) for women with LNG-IUS, endometrial glandular epithelial cells were slightly enlarged with the deeper stained nuclei; the clean background, polar overlap, and homogeneous granular chromatin suggested benign endometrium. And, (5) the endometrial cytological examination by Li Brush was effectively used to screen atypical hyperplasia and carcinoma; however, the endometrial cell smears of women with IUDs were occasionally diagnosed as atypical endometrial cells with undetermined significance; it was necessary to follow up with the combination of medical history and symptoms to determine whether it was due to the copper and exogenous hormone effects or the endometrial lesions.

In this research, we found two advantages of using Li Brush clinically. First, using Li Brush as a supplementary sampling method to D&C was more beneficial for postmenopausal women or patients with local endometrial lesions to increase satisfactory sampling. For example, a postmenopausal patient with hypertension and diabetes was enrolled in the IUD group in January 2020, and the ultrasonographic examination displayed a hypoechoic mass in the uterine cavity. D&C was performed with an unsatisfactory sample. However, the cytological diagnosis by Li Brush was atypical endometrial cells with undetermined significance. A similar case has been reported in our previous research (15). Second, Li Brush can be applied to the follow-up of conservatively treated patients with atypical hyperplasia or early endometrial carcinoma. In this study, a woman using LNG-IUS for conservative treatment of atypical hyperplasia was diagnosed accurately by cytological examination using Li Brush.

There were also some limitations in this study. First, the endometrial thickness of patients in the IUD group was significantly less than that in the control group (*p* < 0.01), which maybe account for medical effects of LNG-IUSs in the IUD group and a higher proportion of endometrial polyps in the control group. Second, no patients with cancer or precancerous lesions were collected in Part II. The patients diagnosed with benign endometrium by D&C and malignant lesions by Li Brush samplers were not further explored for the reasons and followed up. Third, cell block has been used as a complementary technique for increasing diagnostic accuracy in many fields (38, 39). The endometrial specimens sampled by Li Brush can be processed into both cell smears and cell blocks. The micro-histological structures in the cell block were more conducive to the endometrial diagnoses (40). However, the cell block was not used in this study to improve the diagnostic accuracy of Li Brush in endometrial lesions.

## Conclusions

Overall, this research verified that there was no effect on the position of IUDs when Li Brush was used for endometrial sampling. At the same time, Li Brush had a high satisfactory sampling rate for endometrial sampling and good validity for detecting endometrial lesions. Therefore, it is reliable to screen endometrial lesions by Li Brush sampler in women with IUDs.

## Data Availability Statement

The original contributions presented in the study are included in the article/Supplementary Material, further inquiries can be directed to the corresponding author/s.

## Ethics Statement

The studies involving human participants were reviewed and approved by the Ethics Committee of the First Affiliated Hospital of Xi'an Jiaotong University. The patients/participants provided their written informed consent to participate in this study.

## Author Contributions

QL contributed to administrative, research conception, technical and material supports. LH contributed to research conception, data collection, analysis and writing of the manuscript. SM, YL, YW, XF, KZ, PY, and DL contributed to patient recruitment, management, and acquisition of specimens. HH and GS contributed to pathological diagnosis. LiW contributed to the ultrasonography. LZ and LeW contributed to analysis and interpretation of data. All authors saw and approved the final version.

## Conflict of Interest

The authors declare that the research was conducted in the absence of any commercial or financial relationships that could be construed as a potential conflict of interest.
