# Supplementary material for: Clinical Evaluation of Li Brush Endometrial Samplers for Diagnosing Endometrial Lesions in Women With Intrauterine Devices
Source: Front Med (Lausanne). 2020 Nov 30;7:598689. doi: 10.3389/fmed.2020.598689 (PMC7734192; doi:10.3389/fmed.2020.598689)
Supplement: Supplementary file 1 [file Table_1.DOCX]

**Supplementary table 1.** The comparison of diagnosis between cytopathology and histopathology

| Group | endometrial types | Cytopathological diagnoses | | Total |
| --- | --- | --- | --- | --- |
|  |  | accurate | inaccurate |  |
| IUD group | proliferative endometrium | 25 | 6 | 31 |
|  | secretory endometrium | 13 | 1 | 14 |
|  | atrophic endometrium | 1 | 1 | 2 |
|  | mixed endometrium | 3 | 0 | 3 |
|  | hyperplasia without atypia | 39 | 3 | 42 |
|  | Total | 81 | 11 | 92 |
| Control group | proliferative endometrium | 32 | 2 | 34 |
|  | secretory endometrium | 16 | 1 | 17 |
|  | atrophic endometrium | 5 | 1 | 6 |
|  | mixed endometrium | 8 | 1 | 9 |
|  | hyperplasia without atypia | 51 | 3 | 54 |
|  | Total | 112 | 8 | 120 |
