# Supplementary material for: Clinical Evaluation of Li Brush Endometrial Samplers for Diagnosing Endometrial Lesions in Women With Intrauterine Devices
Source: Front Med (Lausanne). 2020 Nov 30;7:598689. doi: 10.3389/fmed.2020.598689 (PMC7734192; doi:10.3389/fmed.2020.598689)
Supplement: Supplementary file 2 [file Table_2.DOCX]

**Supplementary table 2.** The diagnostic accuracy in the subtypes of IUDs

| Subtypes of IUDs | cytopathology and histopathology | | P value |
| --- | --- | --- | --- |
|  | consistent | inconsistent |  |
| OCu200 | 18 | 3 | 0.81 |
| GyneFix IN | 3 | 0 |  |
| MYCu | 29 | 3 |  |
| HCu280 | 2 | 1 |  |
| TCu220c | 4 | 1 |  |
| LNG-IUS | 1 | 0 |  |
| Cu365 | 16 | 3 |  |
| MLCu375 | 4 | 0 |  |
| Others | 4 | 0 |  |
| Total | 81 | 11 |  |
