# Supplementary figures and images for: Clinical Evaluation of Li Brush Endometrial Samplers for Diagnosing Endometrial Lesions in Women With Intrauterine Devices
Source: Front Med (Lausanne). 2020 Nov 30;7:598689. doi: 10.3389/fmed.2020.598689 (PMC7734192; doi:10.3389/fmed.2020.598689)

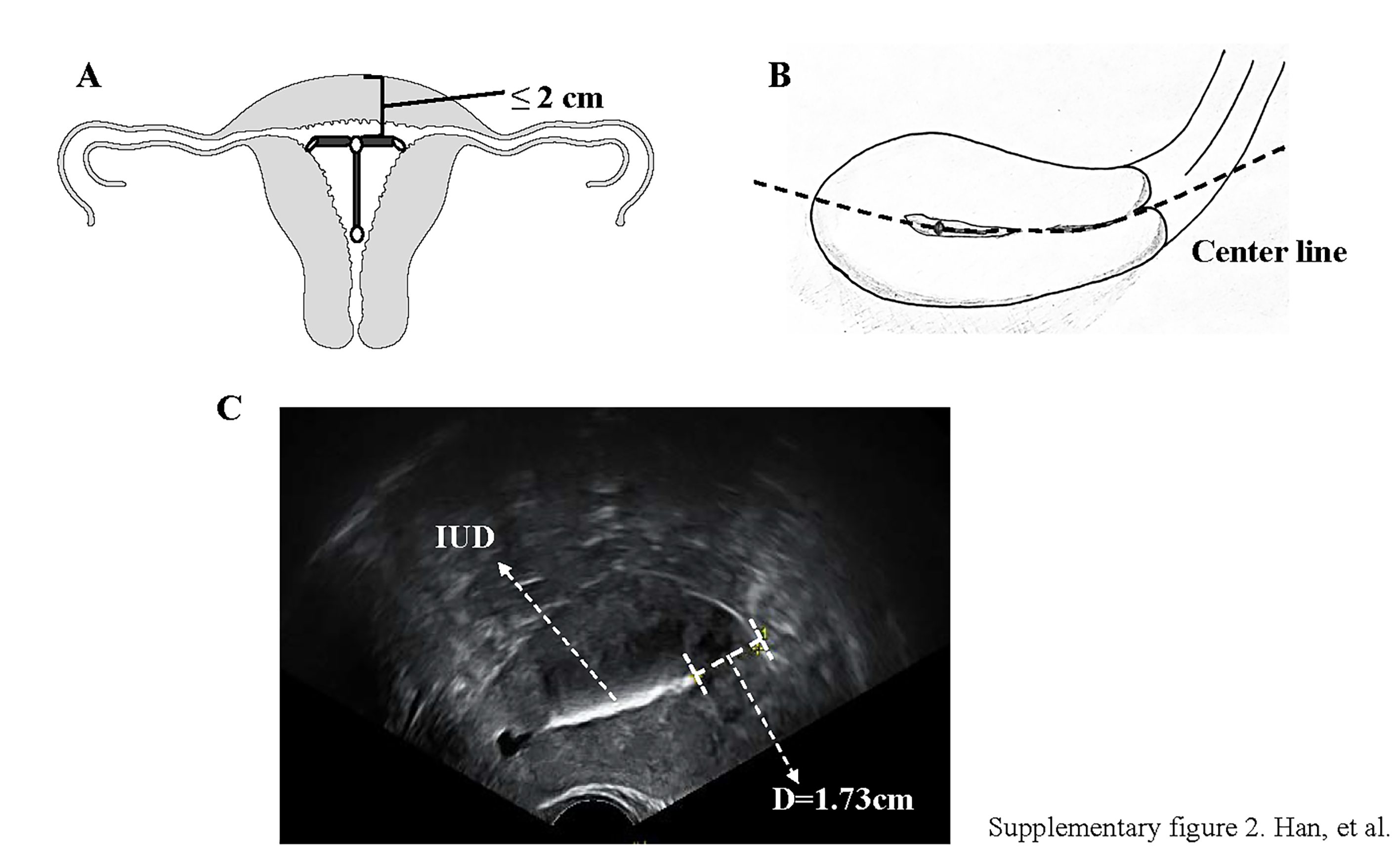

Supplement: Supplementary file 4 [file Image_2.TIFF]
